# Functional Iron-Transport Genes—*TF* and *TMPRSS6*—As Genetic Determinants of Transferrin and Fasting Glucose in a Kazakh Adult Cohort: A Whole-Exome Sequencing Pilot Study

**DOI:** 10.3390/ijms27125374

**Published:** 2026-06-14

**Authors:** Dana Kaldarkhan, Gulnaz Nuskabayeva, Nursultan Nurdinov, Ugilzhan Tatykayeva, Ainash Oshibayeva, Shoira Isanova, Arzu Mamutova, Yusuf Ozkul, Nuriye Gokce, Izem Olcay Sahin, Karlygash Sadykova

**Affiliations:** 1 Faculty of Medicine, Khoja Akhmet Yassawi International Kazakh-Turkish University, Turkestan 160000, Kazakhstan; dana.kaldarkhan2024@ayu.edu.kz (D.K.);; 2 Department of Neurology, Samarkand State Medical University, Samarkand 140103, Uzbekistan; 3 Department of Medical Genetics, School of Medicine, Erciyes University, Kayseri 38039, Turkey

**Keywords:** iron metabolism, metabolic syndrome, TF rs12769, *TMPRSS6* haplotype, transferrin, Kazakh population, candidate-gene association study

## Abstract

Iron metabolism has long been linked to metabolic syndrome (MetS), but it is still unclear at which step—iron sensing, hepcidin regulation, export, transport, or storage—genetic variation matters the most. There are almost no studies on iron metabolism genes in Kazakhs in particular. Using whole-exome sequencing (WES) data from 96 Kazakh adults (52 with MetS), we examined 18 SNPs across six iron metabolism genes—*HFE*, *SLC40A1*, *TMPRSS6*, *FTL*, *TFR2*, and *TF*. Associations with iron biomarkers and MS components were tested by linear regression adjusted for age, sex, and BMI, with FDR correction, haplotype analysis, and bootstrap mediation analysis. Significant effects clustered at two distinct steps of iron metabolism: hepcidin regulation (*TMPRSS6*) and iron transport (*TF*). The T allele of *TF* rs12769 raised serum transferrin (β = +0.32 g/L; p_FDR = 0.002) while lowering both TSAT (β = −4.25%) and ferritin (β = −0.36 log-units); haplotype analysis confirmed rs12769 as the driver. The *TMPRSS6* C–G–C haplotype was associated with lower fasting glucose (β = −1.19 mmol/L; *p* = 0.023), and *TF* rs12769 emerged as a robust FDR-significant determinant of serum transferrin (p_FDR = 0.002). Bootstrap mediation analysis (5000 iterations) showed that the *TMPRSS6* effect on glucose is not mediated by ferritin, serum iron, transferrin, TSAT, or sTfR (all ACME *p* > 0.20), while Total and Direct Effects remained robust (*p* ≤ 0.054). In Kazakhs, iron-metabolism genes appear to influence fasting glucose through direct mechanisms not captured by the standard iron biomarker panel; alternative pathways involving hepatic enzymes, hepcidin, or inflammation warrant investigation in larger cohorts.

## 1. Introduction

Iron is an essential and irreplaceable element in the human body required for oxygen transport, tissue perfusion, DNA synthesis, and various enzymatic processes [1]. In contrast to iron deficiency, excessive iron accumulation may disrupt tissue homeostasis through the generation of reactive oxygen species, lipid peroxidation, and protein modification [2]. Systemic iron homeostasis is regulated by the hepcidin–ferroportin axis, which controls intestinal iron absorption, iron recycling from senescent erythrocytes, and the release of iron from hepatic stores [3].

Recent studies have demonstrated associations between disturbances in iron metabolism and metabolic syndrome (MetS) and its components, including type 2 diabetes mellitus (T2DM), insulin resistance, hypertension, dyslipidemia, and abdominal obesity [4,5]. Several cohort studies have shown that both elevated and high–normal serum ferritin levels are associated with an increased incidence of MetS and T2DM [6,7]. From a pathophysiological perspective, iron overload may impair pancreatic β-cell function through oxidative stress and inflammation, thereby contributing to insulin resistance and adipose tissue dysfunction [8,9]. It should be noted that ferritin is not only a marker of iron stores but also an acute-phase reactant; therefore, its elevation in MetS and T2DM may partly reflect concomitant low-grade systemic inflammation rather than iron dysregulation alone [10]. However, Mendelian randomization studies have yielded inconsistent findings. Earlier studies suggested that elevated systemic iron status may play a causal role in the development of T2DM, whereas more recent bidirectional analyses demonstrated that ferritin, transferrin saturation (TSAT), and total iron-binding capacity (TIBC) do not exert a causal effect on T2DM; the potential positive association of serum iron requires further confirmation [11].

Iron homeostasis is tightly regulated through a highly coordinated network of systemic and cellular mechanisms controlling iron absorption, transport, storage, and recycling [12]. At each of these stages, the genetic architecture of iron homeostasis involves several key regulatory genes. The HFE gene regulates hepcidin production in response to changes in iron status, and pathogenic variants such as C282Y (rs1800562) and H63D (rs1799945) are well-established causes of hereditary hemochromatosis [13]. The *TMPRSS6* gene encodes matriptase-2, a hepatic serine protease that suppresses hepcidin expression by cleaving hemojuvelin. Common *TMPRSS6* variants, including rs855791 and rs4820268, are among the strongest genomic determinants of serum iron, transferrin saturation, ferritin, and hemoglobin levels in both European and Asian populations [14]. The TF gene encodes transferrin, the principal plasma iron transport protein, and contains variants associated with circulating transferrin concentrations [15]. In addition, *TFR2*, *SLC40A1* (ferroportin), and *FTL* (ferritin light chain) genes are involved in iron sensing, export, and storage, respectively [16,17]. Identifying which stages of iron metabolism and which iron-related genes are disrupted in patients with MetS is important for a deeper understanding of the pathophysiological mechanisms underlying the development of metabolic syndrome.

Despite the large number of genetic association studies related to iron metabolism, most investigations have been conducted in populations of European or East Asian ancestry. Central Asian populations, including Kazakhs, remain substantially underrepresented in genomic studies [18]. Located at the intersection of Europe and Asia, the Kazakh population is characterized by considerable genetic admixture, which may contribute to population-specific allele frequencies and effect sizes [19]. Such population-specific data are essential for accurate risk stratification, the development of personalized medicine, and appropriate comparisons across global cohorts.

**Figure 1:**
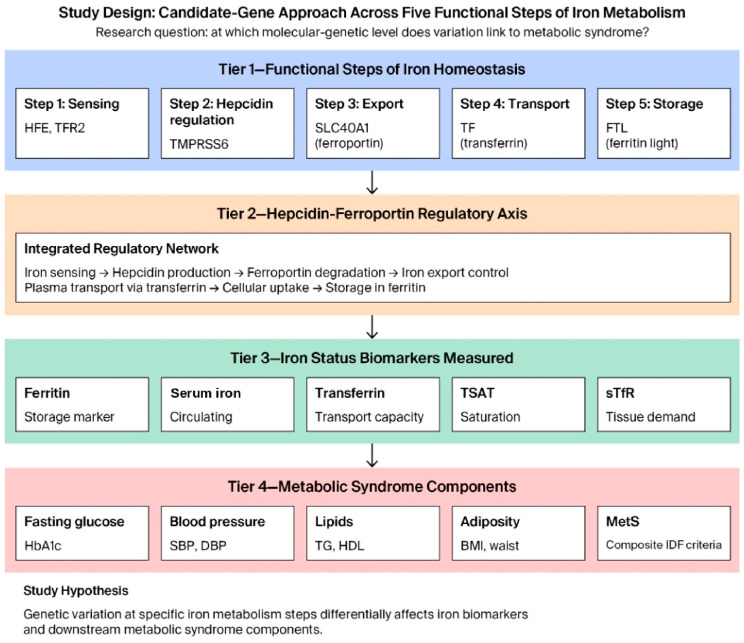
Conceptual framework of the study design. To identify the molecular genetic level at which iron metabolism variability is associated with metabolic syndrome (MetS), we systematically examined 18 SNPs across six candidate genes representing five key functional stages of iron homeostasis (Tier 1): iron sensing (*HFE*, *TFR2*), hepcidin regulation (*TMPRSS6*), iron export (*SLC40A1*), iron transport (*TF*), and iron storage (*FTL*). These pathways converge within the integrated hepcidin–ferroportin regulatory axis (Tier 2), which controls the iron status biomarkers measured in this study (Tier 3), including ferritin, serum iron, transferrin, transferrin saturation (TSAT), and soluble transferrin receptor (sTfR). By examining their associations with MetS components (Tier 4)—fasting glucose, blood pressure, lipid profile, obesity, and the overall MetS diagnosis—we aimed to determine which molecular level plays the predominant role in metabolic alterations related to iron homeostasis.

In this pilot study, leveraging whole-exome sequencing data, we aimed to (i) characterize allele frequencies of 18 SNPs in six genes involved in different stages of iron metabolism (*HFE*, *SLC40A1*, *TMPRSS6*, *FTL*, *TFR2*, and *TF*) in a cohort of adult Kazakhs; (ii) investigate their associations with iron metabolism biomarkers, including ferritin, serum iron, transferrin, transferrin saturation (TSAT), and soluble transferrin receptor (sTfR), as well as with metabolic syndrome and its components; and (iii) formally test, using rigorous bootstrap causal mediation analysis, whether iron biomarkers mediate the relationship between genetic variants and metabolic syndrome and its components (Figure 1).

## 2. Results

### 2.1. Cohort Characteristics

The study included 96 adults of Kazakh ethnicity, comprising 62 women and 34 men. According to the IDF 2009 criteria [20], 52 participants were diagnosed with metabolic syndrome, while the remaining 44 individuals formed the control group. The demographic and clinical characteristics of the participants are presented in Table 1 according to MetS status. Compared with the control group, individuals with metabolic syndrome exhibited statistically significant differences in several parameters. They were older (median age 60.5 vs. 55.0 years; *p* = 0.003), had a larger waist circumference (105.0 vs. 96.0 cm; *p* = 0.005), and higher blood pressure levels (SBP: 130 vs. 110 mmHg, *p* < 0.001; DBP: 80 vs. 70.5 mmHg, *p* = 0.001). In addition, fasting glucose (5.63 vs. 5.06 mmol/L; *p* = 0.014), HbA1c (5.80 vs. 5.65%; *p* = 0.010), and triglyceride levels (1.71 vs. 1.10 mmol/L; *p* = 0.001) were higher, whereas high-density lipoprotein cholesterol (HDL-C) levels were lower (1.13 vs. 1.27 mmol/L; *p* = 0.013). These findings are consistent with the diagnostic components of metabolic syndrome.

Notably, the iron metabolism profile differed selectively between groups. Functional iron transport markers were significantly elevated in MetS: serum transferrin (2.70 vs. 2.50 g/L; *p* = 0.026) and soluble transferrin receptor (sTfR; 3.01 vs. 2.50 mg/L; *p* = 0.008). Transferrin saturation (TSAT) showed a trend toward lower values in MetS (21.0 vs. 26.5%; *p* = 0.098). In contrast, serum ferritin—the principal indicator of iron stores—did not differ between groups (77.1 vs. 83.4 ng/mL; *p* = 0.334), nor did serum iron (15.5 vs. 15.8 µmol/L; *p* = 0.676).

### 2.2. Allele Frequencies and Hardy–Weinberg Equilibrium

**Figure 2:**
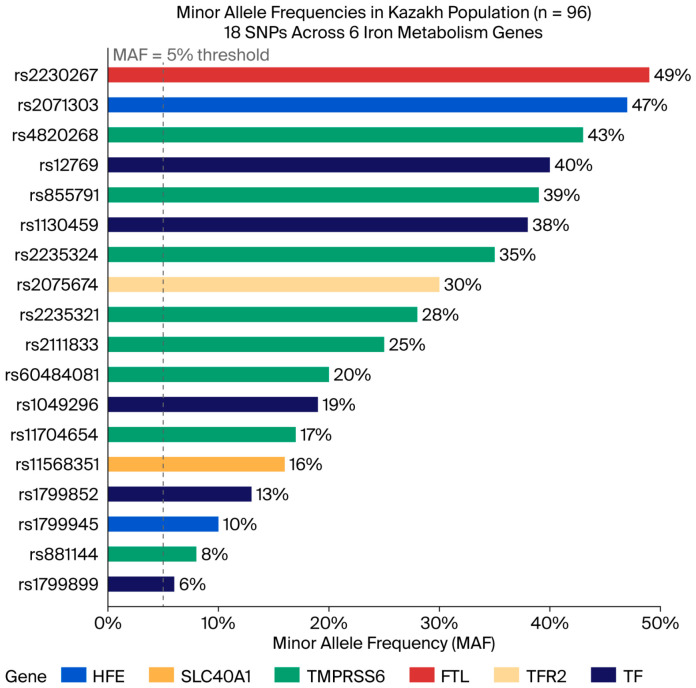
Allele frequencies in the Kazakh population.

From the whole-exome sequencing dataset, minor allele frequencies (MAFs) ranged from 0.062 for *TF* rs1799899 and *FTL* rs2230267 to 0.49 (Supplementary Table S1, Figure 2). All SNPs were in Hardy–Weinberg equilibrium after Bonferroni correction (α = 0.05/18 = 0.0028).

### 2.3. TF rs12769 Is Strongly Associated with Serum Transferrin Levels

**Figure 3:**
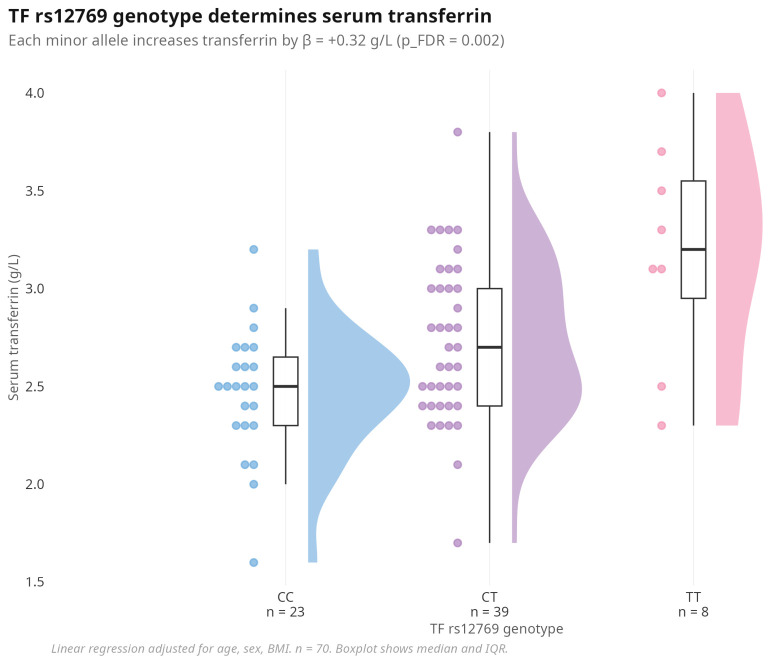
TF rs12769 is strongly associated with serum transferrin levels. Linear regression adjusted for age, sex, and BMI. n = 70. The box plot shows the median and IQR.

Linear regression analysis of 18 SNPs related to iron metabolism and biochemical iron status parameters identified the *TF* rs12769 polymorphism as a robust determinant of serum transferrin levels. Adjustments were made for age, sex, and BMI. Under the additive model, each minor allele of TF rs12769 was associated with an increase in serum transferrin levels (β = +0.32 g/L, 95% CI: 0.16–0.47; *p* = 5.4 × 10^−5^; pFDR = 0.002) (Figure 3). Pleiotropic effects on other iron-related biomarkers were also observed, with each minor allele associated with lower TSAT levels (β = −4.25%; *p* = 0.009) and lower serum ferritin concentrations (β = −0.36 log-units; *p* = 0.027). These findings are consistent with physiological changes related to increased plasma iron-binding capacity.

Among the five *TF* gene polymorphisms analyzed (rs1799852, rs1799899, rs1049296, rs1130459, and rs12769), haplotype analysis further supported the central role of rs12769. Three haplotypes carrying the C allele at the rs12769 position were associated with lower transferrin levels compared with the reference haplotype (β ranging from −0.27 to −0.52 g/L; all *p* ≤ 0.010). In contrast, haplotypes lacking the T allele at rs12769, but differing at the remaining four SNPs, did not significantly deviate from the reference. These findings were consistent with the single-SNP analysis, in which each T allele was associated with a +0.34 g/L increase in transferrin levels, supporting rs12769 as the principal determinant of transferrin concentrations within the analyzed SNP panel (Figure 3).

### 2.4. No FDR-Significant Associations Were Identified with Metabolic Syndrome Status

**Figure 4:**
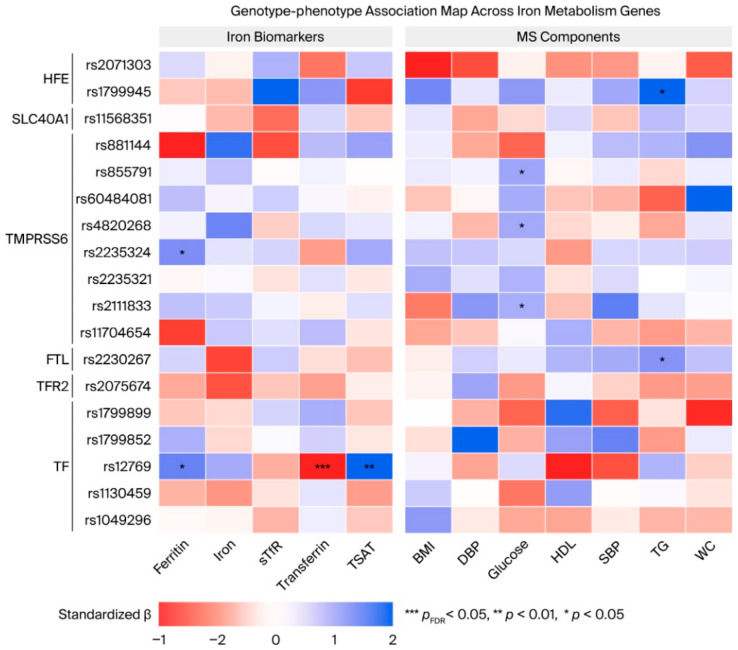
Genotype–phenotype association map.

Logistic regression analysis examining associations between iron metabolism-related polymorphisms and metabolic syndrome components revealed no FDR-significant associations after adjustment for age and sex (Table S2, Figure 4).

### 2.5. TMPRSS6 Haplotype Is Associated with Lower Fasting Glucose Levels

Several *TMPRSS6* polymorphisms showed inverse associations with fasting glucose levels under the additive model (adjusted for age, sex, and BMI): rs855791 (β = −0.95 mmol/L; *p* = 0.021), rs4820268 (β = −0.91; *p* = 0.028), rs2111833 (β = −0.88; *p* = 0.042)**,** and rs2235321 (β = −0.82; *p* = 0.064). Although these individual SNP associations did not remain significant after FDR correction, they demonstrated a consistent joint pattern.

To strengthen the overall signal, haplotype analysis was performed for three functional *TMPRSS6* polymorphisms: rs855791, rs4820268, and rs2111833. Compared with carriers of the reference haplotype (T–A–T), carriers of the minor haplotype C–G–C (frequency 18.9%) showed significantly lower fasting glucose levels (β = −1.19 mmol/L; *p* = 0.023). A second minor haplotype, C–G–T (frequency 19.6%), demonstrated a similar trend (β = −1.02; *p* = 0.054).

**Figure 5:**
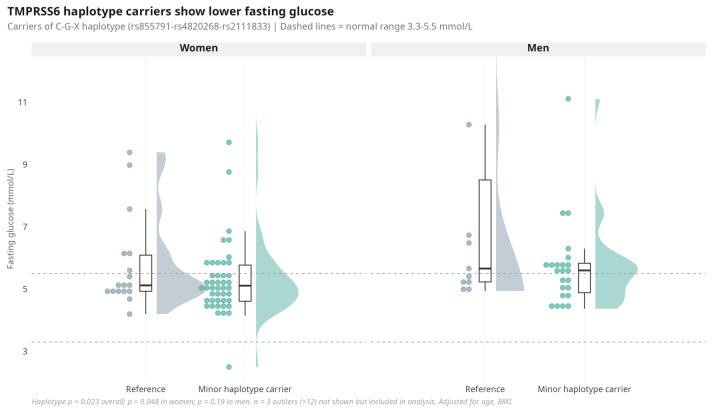
Association of *TMPRSS6* haplotype with fasting glucose, stratified by sex. Adjusted for age.

Sex-stratified analysis showed a consistent direction of effect in both groups. Among women (n = 62), the *TMPRSS6* C–G–C haplotype was significantly associated with lower glucose levels (β = −0.79; *p* = 0.048), whereas in men (n = 34) the effect size was substantially larger (β ranging from −2.10 to −2.80) but did not reach statistical significance due to the limited sample size (Figure 5).

### 2.6. Causal Mediation Analysis: TMPRSS6 → Fasting Glucose

To test whether iron biomarkers mediated the *TMPRSS6* association with fasting glucose, we performed bootstrap causal mediation analysis (5000 iterations) with *TMPRSS6* rs4820268 (additive coding) as the exposure, fasting glucose as the outcome, and ferritin, serum iron, transferrin, TSAT, and sTfR as candidate mediators in separate single-mediator models adjusted for age, sex, and BMI.

The Total Effect of *TMPRSS6* on glucose was significant across all models (Table 2): β = −0.91 mmol/L per minor allele (*p* = 0.002) in the ferritin model (n = 96), and β = −0.64 (*p* = 0.038) in the other models (n = 71). The Average Direct Effect (ADE) was also significant (*p* = 0.001–0.054), whereas the Average Causal Mediation Effect (ACME) was null for every biomarker—all *p*-values exceeded 0.20, and 95% confidence intervals widely crossed zero.

A joint mediation model in lavaan, including all four functional biomarkers (iron, transferrin, TSAT, sTfR) as parallel mediators, confirmed the same pattern: a significant standardized direct effect (c = −0.22; *p* = 0.042) and total effect (−0.20; *p* = 0.046), but a null combined indirect effect (0.020; *p* = 0.67).

**Figure 6:**
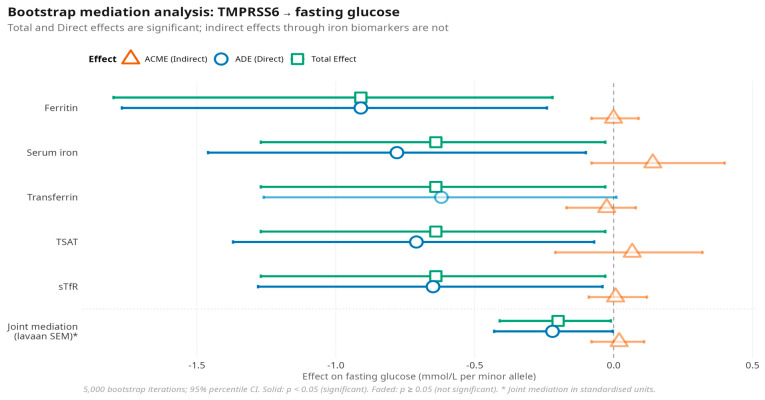
Bootstrap mediation analysis.

Taken together (Figure 6), the *TMPRSS6* → fasting glucose association in this Kazakh pilot cohort is best characterized as a direct genetic effect, not mediated by measured iron biomarkers.

## 3. Discussion

In this pilot study conducted in a Kazakh population, we performed a systematic analysis of 18 polymorphisms in six iron metabolism genes and their associations with iron biomarkers, metabolic syndrome, and its components. At the descriptive stage of the cohort analysis, we observed a selective increase in functional iron transport markers—transferrin and soluble transferrin receptor—among individuals with MetS, whereas ferritin levels did not differ significantly between groups. This observation proved to be biologically consistent with the two principal findings of our association analyses.

Our study yielded two main findings. First, we identified *TF* rs12769 as a robust determinant of serum transferrin levels in the Kazakh population and demonstrated its pleiotropic effects on TSAT and ferritin concentrations. Second, a three-SNP *TMPRSS6* haplotype was associated with lower fasting glucose levels, and rigorous bootstrap mediation analysis showed that this effect operates as a direct genetic effect, not mediated by measured iron biomarkers (ferritin, serum iron, transferrin, TSAT, or sTfR).

Functional biomarkers of iron metabolism (transferrin, TSAT, and sTfR) reflect the actively circulating iron fraction available for erythropoiesis and cellular metabolism and are more sensitive than storage markers to early alterations in systemic iron homeostasis [21]. In contrast, ferritin is not only a marker of tissue iron stores but also an acute-phase protein whose plasma concentration is substantially elevated in the presence of low-grade systemic inflammation, metabolic syndrome, and hepatic steatosis—conditions commonly observed in the study cohort [10]. Therefore, genetic effects detected specifically through functional iron biomarkers (as in the case of *TF* rs12769 in our study) may more reliably reflect direct mechanisms of iron regulation, whereas ferritin-related signals require additional interpretation in light of potential inflammatory influences [22].

### 3.1. TF rs12769 as a Determinant of Plasma Transferrin

The strong association between *TF* rs12769 and transferrin levels (pFDR = 0.002) supports the biological relevance of the *TF* locus in iron metabolism. In the study by Bell S. et al., which combined UK Biobank and deCODE Genetics data (n > 200,000), TF rs12769 was identified as one of the principal determinants of serum transferrin concentration with a consistent direction of effect across all analyzed populations [23]. In addition, genome-wide association studies by Benyamin B. et al. and McLaren C.E. et al. demonstrated that variants within the *TF* locus and its regulatory regions are associated with circulating transferrin concentrations in European populations [15,24]. Our findings are consistent with these reference studies and extend the evidence to the Kazakh population.

### 3.2. TMPRSS6 and Glucose Homeostasis

Iron accumulation in tissues has been associated with insulin resistance, impaired pancreatic β-cell function, and an increased risk of T2DM through Fenton reaction-dependent oxidative stress mechanisms [4,5,6,7]. *TMPRSS6* encodes matriptase-2, a transmembrane serine protease expressed in hepatocytes that negatively regulates hepcidin transcription through cleavage of hemojuvelin. Minor alleles of rs855791 (T>C, Ala736Val) and rs4820268 (A>G) are considered loss-of-function variants that reduce the proteolytic activity of matriptase-2, leading to increased hepcidin production, ferroportin inhibition, and lower systemic iron bioavailability [14,25]. As a consequence, fasting glucose levels may decrease, and the risk of T2DM may be reduced. Large cohort study has similarly shown that carriers of these variants exhibit lower transferrin saturation, lower serum iron levels, elevated sTfR, and a higher prevalence of iron deficiency anemia [26].

Thus, the *TMPRSS6* minor haplotype may represent a genetically determined mild hypoferremic phenotype that, in our study, demonstrated a protective metabolic effect. Our findings are also consistent with Mendelian randomization studies reporting that genetically determined lower iron status is causally associated with reduced T2DM risk. In particular, MR studies using *TMPRSS6* variants as instrumental variables demonstrated that lower serum iron predicts reduced T2DM risk in European populations [27]. It is important to distinguish two distinct phenomena: while elevated iron status is associated with increased risk of incident T2DM, established T2DM frequently coexists with functional iron-deficiency anemia driven by chronic inflammation and hepcidin dysregulation [28]. These are not contradictory but complementary mechanisms operating at different stages of disease progression.

Sex-stratified analysis revealed a consistent direction of effect in both subgroups: in women (n = 62), the TMPRSS6 C–G–C haplotype was significantly associated with lower glucose levels (β = −0.79; *p* = 0.048), whereas in men (n = 34) the effect size was substantially larger (β ranging from −2.10 to −2.80) but did not reach statistical significance. This pattern aligns with known sex differences in iron homeostasis—men have higher baseline iron stores and a stronger hepcidin-mediated response—suggesting that *TMPRSS6* variants may exert a more pronounced metabolic effect in male carriers, which warrants confirmation in larger sex-balanced cohorts.

To the best of our knowledge, this is the first study to (1) confirm the protective role of the *TMPRSS6* haplotype at the individual carrier level in a Central Asian cohort, and (2) formally evaluate iron biomarker mediation using rigorous bootstrap causal methods, demonstrating that this effect operates through direct pathways rather than measured iron pools.

### 3.3. Associations of HFE and FTL with Triglycerides

Beyond the main findings, we observed nominal associations between *HFE* and *FTL* polymorphisms and triglyceride (TG) levels that did not survive FDR correction but remain biologically interesting. Both genes belong to the storage iron compartment: *HFE* regulates iron uptake by hepatocytes through hepcidin [29], while *FTL* encodes the ferritin light chain [30]. Hepatic iron accumulation has been described as a driver of de novo lipogenesis and elevated circulating TG in hepatic steatosis and hereditary haemochromatosis [31]. In this context, our findings are biologically plausible, though confirmation in a larger sample with concurrent assessment of steatosis and hepatic function is required—a priority of our ongoing extension study.

### 3.4. TMPRSS6 Influences Fasting Glucose Through Pathways Beyond the Standard Iron Panel

We initially hypothesized that the *TMPRSS6* → fasting glucose association (Total Effect *p* = 0.002) would be mediated by functional iron-transport biomarkers, consistent with the canonical role of *TMPRSS6* (matriptase-2) as a negative regulator of hepcidin transcription [25] and prior GWAS findings linking *TMPRSS6* variants to iron homeostasis and metabolic traits [26]. However, formal bootstrap mediation analyses—including single-mediator, joint SEM, and sex-stratified models—did not support this hypothesis: no measured iron biomarker mediated the relationship significantly, while the Average Direct Effect remained robust (*p* = 0.001–0.054). The genetic effect on glucose is real but operates through pathways other than the standard iron panel.

Three candidate pathways deserve attention. First, hepatic dysfunction: *TMPRSS6* is predominantly expressed in hepatocytes, where it may directly affect gluconeogenesis and insulin clearance independently of iron flux; hepatic enzymes such as GGT, ALT, and AST—established predictors of incident type 2 diabetes [32]—were not measured in the present pilot. Second, circulating hepcidin—the proximal molecular target of *TMPRSS6*—would be the most direct intermediate but requires specialized immunoassay. Third, low-grade inflammation (hs-CRP) is bidirectionally linked to both iron metabolism and glucose dysregulation.

### 3.5. Population-Specific Allele Frequencies

To the best of our knowledge, this is the first report describing allele frequencies of 18 iron metabolism-related SNPs in a Kazakh cohort. Most allele frequencies were within European reference ranges; however, *TMPRSS6* rs2111833 and *TF* rs1130459 showed lower frequencies than European and East Asian populations, which is expected for Central Asian populations with mixed genetic ancestry [19]. *TMPRSS6* rs2111833 in the Kazakh cohort (MAF = 0.250) showed a lower frequency compared to both East Asian (EAS, MAF = 0.336) and European (NFE, MAF = 0.339) reference populations. A similar pattern was observed for *TF* rs1130459 (Kazakh MAF = 0.380), with a frequency markedly lower than both European (NFE, MAF = 0.527) and East Asian (EAS, MAF = 0.759) populations [33]. These reference data may contribute to future risk stratification and personalized medicine efforts in Kazakhstan and neighboring countries.

### 3.6. Strengths and Limitations

A major strength of our study is the use of whole-exome sequencing for genotyping, providing high-quality direct sequencing data rather than imputed genotypes. This approach also enables future exploration of additional variants in iron metabolism genes beyond the candidate SNPs analyzed here. Another strength is the expanded panel of iron metabolism biomarkers (n = 71 with complete transferrin, serum iron, TSAT, and sTfR data), the use of three complementary genetic approaches (single-SNP additive analysis, haplotype analysis, and mediation analysis), FDR correction, and the focus on a previously underrepresented population. Several limitations should also be acknowledged: The sample size provided adequate power for the primary direct effects (≈80% for *TMPRSS6* → glucose Total Effect at standardized |β| ≥ 0.30), the pilot was under-powered for detecting small indirect (mediation) effects of the observed magnitude (point estimates < |0.15|), which would require approximately n = 300–500 participants. The detection of binary outcomes (MetS as a whole) and variants with MAF < 0.10 was similarly limited. The stronger effect of *TMPRSS6* on glucose observed in men did not reach statistical significance and requires confirmation in larger cohorts. The cross-sectional design precludes definitive causal conclusions, although the direction of our findings is consistent with previous Mendelian randomization studies [11]. Population-specific effects should be replicated in independent Central Asian cohorts. Finally, hepcidin levels were not measured, and inclusion of this biomarker would enable more direct validation of the proposed mechanistic pathway.

## 4. Materials and Methods

### 4.1. Ethical Approval and Informed Consent

After obtaining approval from the ethics committee, the participants were provided with a clear and simple explanation of the purpose of the study, the study procedures, and the potential risks and benefits. Additional explanations were provided to ensure that each participant fully understood what would be required during the study and his or her role in it. After asking questions and receiving complete answers, the participants provided written informed consent by signing the informed consent form.

### 4.2. Anthropometric, Biochemical, and Clinical Data

Participants were recruited from the clinic of Khoja Akhmet Yassawi International Kazakh–Turkish University in Turkistan. Potential participants were contacted through general practitioners and invited to take part in the study. Patients who provided informed consent were referred for physical examination and blood sample collection. Physical measurements included height, body weight, waist circumference, and hip circumference. Height was measured using a stadiometer. Body weight was measured using an electronic scale. After height and body weight were determined, body mass index (BMI) was calculated as weight (kg) divided by height squared (m^2^). Waist circumference was measured using a flexible measuring tape with an accuracy of 0.1 cm. Blood samples were collected into serum separation tubes and tubes containing ethylenediaminetetraacetic acid (EDTA).

In this study, metabolic syndrome (MetS) was defined according to the criteria of the International Diabetes Federation (IDF, 2009) [20]. MetS was diagnosed when three or more of the following five criteria were present: triglycerides ≥ 1.7 mmol/L; high-density lipoprotein cholesterol (HDL-C) < 1.03 mmol/L in men and <1.29 mmol/L in women; blood pressure ≥ 130/85 mmHg; fasting glucose ≥ 5.6 mmol/L; and waist circumference > 90 cm in men and >80 cm in women.

### 4.3. DNA Isolation

In this study, peripheral whole blood samples collected from participants were used for genomic DNA extraction. Genomic DNA was isolated using the GeneAll DNA Extraction Kit (GeneAll Biotechnology Co., Ltd., Seoul, Republic of Korea) according to the manufacturer’s protocol. The kit is based on an SV spin-column system designed for genomic DNA isolation from blood and body fluids.

### 4.4. Whole Exome Sequencing (WES) and Bioinformatics Analysis

DNA libraries were prepared according to standard manufacturer protocols, including random fragmentation, adapter ligation, and PCR amplification steps. Target exonic regions were enriched using the Twist Human Comprehensive Exome Kit (Twist Bioscience, San Francisco, CA, USA). Sequencing was performed on the Illumina NovaSeq 6000 platform (Illumina, San Diego, CA, USA) using a paired-end approach, generating raw FASTQ files.

Alignment, variant calling, and annotation were conducted using the clinically validated and CE-IVD–certified SOPHiA DDM™ v5.4.1 platform (Sophia Genetics SA, Rolle, Switzerland). The GRCh37/hg19 reference genome was used for all analyses. This reference assembly was selected because, at the time of the study, the SOPHiA DDM™ analytical workflow, including its variant-calling algorithms, clinical validation framework, and annotation pipelines, had been optimized and validated using GRCh37/hg19. The use of this reference genome ensured analytical consistency, preserved data integrity, and maintained compatibility with clinically relevant variant annotations from major databases, including ClinVar and dbSNP.

Variants were filtered according to the following sequential criteria. Variants with a sequencing depth of ≥10× and a variant allele fraction of ≥30% were retained. Exonic variants and splice-site variants located within 10 nucleotides of exon–intron boundaries were included, whereas synonymous, untranslated region (UTR), intergenic, and upstream/downstream variants were excluded. Common variants with a minor allele frequency (MAF) > 0.1% in gnomAD, the 1000 Genomes Project, ESP6500, and the SOPHiA DDM database were filtered out. Missense variants predicted as benign by the majority of in silico prediction tools, including SIFT, PolyPhen-2, and MutationTaster, were also excluded. Priority was given to phenotype-related genes, and additional filters were applied according to inheritance models (autosomal dominant, autosomal recessive, and X-linked) as well as de novo occurrence. Clinical pathogenicity classification was performed according to the guidelines of the American College of Medical Genetics and Genomics.

### 4.5. Variant Filtering and Interpretation

Variants obtained after the bioinformatics analyses with a read depth of ≥10 and allele fraction of ≥30% were included. Protein-coding exonic variants and intronic variants within ±10 nucleotides from the exon-intron boundary were included in the analysis, synonymous variants, variants located in non-coding regions, intergenic, upstream and downstream variants were excluded. Common variants with a minor allele frequency (MAF) below 0.1% in population databases such as gnomAD, ExAC, 1000 Genomes, dbSNP and ESP6500 and the SOPHiA DDM (version 7.19.0.2-h853950-9adac33; pipeline revision v5.5.) internal database were evaluated. Pathogenicity classification was performed using in silico analysis tools such as SIFT, PolyPhen-2, GERP and MutationTaster. Variants were comparatively analyzed using databases such as ClinVar, Franklin, Varsome and HGMD and relevant scientific publications. The identified variants were classified according to the American College of Medical Genetics and Genomics (ACMG) guidelines.

### 4.6. Statistical Analysis

All analyses were done in R 4.5.3 with Hardy–Weinberg (1.7.9), haplo.stats (1.9.8.7), tidyverse (2.0.0), ggplot2 (4.0.2), and ggdist (3.3.3). Two-sided *p* < 0.05 was nominal; FDR values are flagged. Continuous data are shown as mean ± SD or median (IQR), depending on the distribution. Group comparisons used *t*-tests, Mann–Whitney *U*, or χ^2^/Fisher tests. Hardy–Weinberg equilibrium was checked in the non-MS controls with Bonferroni correction. SNPs with MAF below 5% were excluded—too few carriers.

Single-SNP associations were tested by linear regression under an additive model (0/1/2 minor alleles), adjusting for age, sex, and BMI. Ferritin and triglycerides were log-transformed. Binary outcomes were modeled with logistic regression. We applied the Benjamini–Hochberg FDR within each phenotype family.

Haplotypes for *TF* (5 SNPs) and *TMPRSS6* (3 SNPs) were reconstructed and tested with haplo.stats using the most common haplotype as reference.

A priori power calculations indicated approximately 80% power to detect a *TMPRSS6* → glucose Total Effect of standardized |β| ≥ 0.30 at α = 0.05, with more limited power (55–65%) for indirect effects of small-to-medium magnitude. Causal mediation analysis was performed using the R package *mediation* (4.5.1) [34] with 5000 nonparametric bootstrap iterations and 95% percentile confidence intervals for ACME, ADE, Total Effect, and Proportion Mediated. A joint mediation model was additionally fitted via structural equation modeling *lavaan*, (0.6-21) [35] with bootstrap standard errors, and analyses were repeated separately in men and women.

## 5. Conclusions

In this pilot study of a Kazakh population, *TF* rs12769 emerged as a stable determinant of serum transferrin levels, while the *TMPRSS6* rs855791–rs4820268-rs2111833 haplotype was associated with lower fasting glucose levels. Rigorous bootstrap mediation analysis revealed that the *TMPRSS6* effect on fasting glucose is not mediated by the standard iron biomarker panel (ferritin, serum iron, transferrin, TSAT, sTfR), indicating a direct genetic effect operating through pathways yet to be characterized.

## Figures and Tables

**Table 1:** Characteristics of study participants by metabolic syndrome status.

Characteristic	Overall ^1^ (N = 96)	Control ^1^ (N = 44)	MetS ^1^ (N = 52)	*p*-Value ^2^
Age, years	58.0 (50.0, 64.3)	55.0 (48.0, 60.0)	60.5 (52.0, 67.3)	0.003
Sex, n (%)				0.187
Female	62 (64.6)	32 (72.7)	30 (57.7)	
Male	34 (35.4)	12 (27.3)	22 (42.3)	
BMI, kg/m^2^	30.1 (27.8, 33.6)	29.9 (26.3, 33.6)	30.3 (28.6, 33.6)	0.308
BMI category, n (%)				0.103
Normal (<25)	7 (9.3)	5 (16.7)	2 (4.4)	
Overweight (25–30)	29 (38.7)	13 (43.3)	16 (35.6)	
Obese (≥30)	39 (52.0)	12 (40.0)	27 (60.0)	
Waist circumference, cm	101.5 (96.0, 110.0)	96.0 (90.3, 104.8)	105.0 (100.0, 111.0)	0.005
SBP, mmHg	120.0 (110.0, 130.3)	110.0 (107.3, 120.0)	130.0 (110.0, 140.0)	<0.001
DBP, mmHg	80.0 (70.0, 80.0)	70.5 (70.0, 80.0)	80.0 (75.8, 90.0)	0.001
Fasting glucose, mmol/L	5.23 (4.85, 5.89)	5.06 (4.79, 5.41)	5.63 (4.92, 6.34)	0.014
HbA1c, %	5.80 (5.50, 6.17)	5.65 (5.40, 5.88)	5.80 (5.68, 6.34)	0.010
Triglycerides, mmol/L	1.44 (1.02, 1.95)	1.10 (0.87, 1.71)	1.71 (1.30, 2.01)	0.001
HDL-C, mmol/L	1.17 (0.98, 1.50)	1.27 (1.07, 1.64)	1.13 (0.93, 1.27)	0.013
Ferritin, ng/mL	79.7 (30.6, 121.9)	83.4 (38.0, 130.2)	77.1 (25.3, 117.9)	0.334
Serum iron, µmol/L	15.5 (11.5, 18.8)	15.8 (11.9, 17.7)	15.5 (11.2, 18.9)	0.676
Transferrin, g/L	2.60 (2.40, 3.00)	2.50 (2.30, 2.70)	2.70 (2.50, 3.05)	0.026
TSAT, %	22.0 (17.0, 28.0)	26.5 (17.0, 32.3)	21.0 (17.5, 27.0)	0.098

1. Median (Q1, Q3); n (%). 2. Wilcoxon rank sum test; Fisher’s exact test.

**Table 2:** Bootstrap causal mediation analysis. *TMPRSS6* rs4820268 (additive) → fasting glucose, by each iron biomarker mediator. ACME = Average Causal Mediation Effect; ADE = Average Direct Effect. All models adjusted for age, sex, and BMI. Bootstrap iterations: 5000.

Mediator	n	ACME (95% CI)	ACME *p*	ADE (95% CI)	Total (95% CI)	Total *p*
Ferritin	96	−0.0004 (−0.08, 0.09)	0.97	−0.91 (−1.77, −0.24)	−0.91 (−1.80, −0.22)	0.002
Serum iron	71	0.141 (−0.08, 0.40)	0.20	−0.78 (−1.46, −0.10)	−0.64 (−1.27, −0.03)	0.038
Transferrin	71	−0.025 (−0.17, 0.08)	0.65	−0.62 (−1.26, 0.01)	−0.64 (−1.27, −0.03)	0.038
TSAT	71	0.067 (−0.21, 0.32)	0.56	−0.71 (−1.37, −0.07)	−0.64 (−1.27, −0.03)	0.038
sTfR	71	0.007 (−0.09, 0.12)	0.91	−0.65 (−1.28, −0.04)	−0.64 (−1.27, −0.03)	0.038
Joint mediation	71	0.020 (−0.08, 0.11)	0.67	0.22 (−0.43, −0.003)	−0.20 (−0.41, −0.01)	0.046

## Data Availability

The data presented in this study are not publicly available due to ethical and privacy restrictions related to whole-exome sequencing and clinical data.

## Supplementary Materials

The following supporting information can be downloaded at: https://www.mdpi.com/article/10.3390/ijms27125374/s1.
