# Supplementary material for: Functional Iron-Transport Genes—TF and TMPRSS6—As Genetic Determinants of Transferrin and Fasting Glucose in a Kazakh Adult Cohort: A Whole-Exome Sequencing Pilot Study
Source: Int J Mol Sci. 2026 Jun 14;27(12):5374. doi: 10.3390/ijms27125374 (PMC13299320; doi:10.3390/ijms27125374)
Supplement: Supplementary file 1 [file ijms-27-05374-s001.zip › ijms-4364418-supplementary.pdf]

Supplementary Materials

"Functional Iron-Transport Genes — TF and TMPRSS6 — as Genetic Determinants of Transferrin and Fasting Glucose in a Kazakh Adult Cohort: A Whole-Exome Sequencing Pilot Study"

Dana Kaldarkhan[1], Gulnaz Nuskabayeva[1], Nursultan Nurdinov[1], Ugilzhan Tatykayeva[1], Ainash Oshibayeva[1], Shoira Isanova[2], Arzu Mamutova[1], Yusuf Ozkul[3], Nuriye Gokce[3], Izem Olcay Sahin[3], and Karlygash Sadykova[1]

[1] *Khoja Akhmet Yassawi International Kazakh-Turkish University, Turkestan 160000, Kazakhstan;*

[2] *Department of Neurology, Samarkand State Medical University, Samarkand 140103, Uzbekistan;*

[3] *Department of Medical Genetics, School of Medicine, Erciyes University, Kayseri 38039, Turkey*

Table S1. Allele frequencies and Hardy–Weinberg equilibrium

Minor allele frequencies (MAF) and genotype counts for 18 SNPs in six iron metabolism genes in the Kazakh cohort (n = 96). HFE rs1800562 (C282Y) was not detected in the cohort, consistent with its European-specific distribution. Reference frequencies for European (NFE) and East Asian (EAS) populations are provided from the ALFA database (NCBI dbSNP) for comparison.

SNP	n	Genotypes	Minor allele	Kazakh MAF	NFE MAF	EAS MAF
HFE rs2071303	95	CC=17, TC=56, TT=22	T	0.474	—	—
HFE rs1799945 (H63D)	95	CC=75, CG=20	G	0.105	0.137	0.029
SLC40A1 rs11568351	96	CC=70, CG=22, GG=4	G	0.156	0.05	0.30
TMPRSS6 rs60484081	96	AA=58, AB=38	B	0.198	0.27	0.12
TMPRSS6 rs2235321	96	CC=49, CT=41, TT=6	T	0.276	0.50	0.45
TMPRSS6 rs4820268	96	AA=28, AG=53, GG=15	G	0.432	0.45	0.55

TMPRSS6 rs855791	96	CC=13, TC=49, TT=34	C	0.391	0.45	0.55
TMPRSS6 rs2235324	96	CC=42, CT=40, TT=14	T	0.354	0.50	0.45
TMPRSS6 rs2111833	96	CC=7, TC=34, TT=55	C	0.250	0.339	0.336
TMPRSS6 rs881144	96	AA=81, AG=15	G	0.078	0.20	0.05
TMPRSS6 rs11704654	96	CC=70, CT=20, TT=6	T	0.167	0.40	0.20
FTL rs2230267	96	CC=24, CT=46, TT=26	C	0.490	0.40	0.50
TFR2 rs2075674	95	AA=51, AG=30, GG=14	G	0.305	0.35	0.50
TF rs1799852	96	CC=72, CT=23, TT=1	T	0.130	0.10	0.10
TF rs1799899	96	CC=85, CT=10, TT=1	T	0.062	0.15	0.05
TF rs1049296	96	CC=63, CT=29, TT=4	T	0.193	0.20	0.40
TF rs1130459	96	CC=13, CT=47, TT=36	C	0.380	0.527	0.759
TF rs12769	95	CC=32, CT=51, TT=12	T	0.395	0.45	0.55

Table S2. Genotype distributions by metabolic syndrome status

Genotype counts (n, %) for each of 18 SNPs stratified by metabolic syndrome (MetS) status, classified according to the IDF 2009 criteria. Total n = 96 (controls n = 44, MetS n = 52) following correction of HDL units. p-values from Pearson's Chi-squared test or Fisher's exact test (where expected counts < 5). No SNP showed a statistically significant association with MetS status, consistent with the focus of the main analysis on individual MetS components (transferrin, glucose, triglycerides) rather than the binary MetS outcome.

SNP	Controls (n = 44)	MetS (n = 52)	p-value
HFE rs2071303	CC: 9 (17%) | TC: 32 (60%) | TT: 12 (23%)	CC: 8 (19%) | TC: 24 (57%) | TT: 10 (24%)	>0.9
HFE rs1799945	CC: 42 (79%) | CG: 11 (21%)	CC: 33 (79%) | CG: 9 (21%)	>0.9
SLC40A1 rs11568351	CC: 40 (74%) | CG: 11 (20%) | GG: 3 (6%)	CC: 30 (71%) | CG: 11 (26%) | GG: 1 (2%)	0.6
TMPRSS6 rs60484081	AA: 31 (57%) | AB: 23 (43%)	AA: 27 (64%) | AB: 15 (36%)	0.5
TMPRSS6 rs2235321	CC: 25 (46%) | CT: 25 (46%) | TT: 4 (7%)	CC: 24 (57%) | CT: 16 (38%) | TT: 2 (5%)	0.6
TMPRSS6 rs4820268	AA: 15 (28%) | AG: 29 (54%) | GG: 10 (19%)	AA: 13 (31%) | AG: 24 (57%) | GG: 5 (12%)	0.7
TMPRSS6 rs855791	CC: 9 (17%) | TC: 27 (50%) | TT: 18 (33%)	CC: 4 (9%) | TC: 22 (52%) | TT: 16 (38%)	0.6
TMPRSS6 rs2235324	CC: 26 (48%) | CT: 19 (35%) | TT: 9 (17%)	CC: 16 (38%) | CT: 21 (50%) | TT: 5 (12%)	0.3
TMPRSS6 rs2111833	CC: 4 (7%) | TC: 18 (33%) | TT: 32 (59%)	CC: 3 (7%) | TC: 16 (38%) | TT: 23 (55%)	0.9
TMPRSS6 rs881144	AA: 46 (85%) | AG: 8 (15%)	AA: 35 (83%) | AG: 7 (17%)	0.8
TMPRSS6 rs11704654	CC: 40 (74%) | CT: 12 (22%) | TT: 2 (4%)	CC: 30 (71%) | CT: 8 (19%) | TT: 4 (10%)	0.6

FTL rs2230267	CC: 13 (24%) ǀ CT: 25 (46%) ǀ TT: 16 (30%)	CC: 11 (26%) ǀ CT: 21 (50%) ǀ TT: 10 (24%)	0.8
TFR2 rs2075674	AA: 31 (58%) ǀ AG: 15 (28%) ǀ GG: 7 (13%)	AA: 20 (48%) ǀ AG: 15 (36%) ǀ GG: 7 (17%)	0.6
TF rs1799852	CC: 40 (74%) ǀ CT: 14 (26%) ǀ TT: 0 (0%)	CC: 32 (76%) ǀ CT: 9 (21%) ǀ TT: 1 (2%)	0.6
TF rs1799899	CC: 47 (87%) ǀ CT: 7 (13%) ǀ TT: 0 (0%)	CC: 38 (90%) ǀ CT: 3 (7%) ǀ TT: 1 (2%)	0.3
TF rs1049296	CC: 33 (61%) ǀ CT: 19 (35%) ǀ TT: 2 (4%)	CC: 30 (71%) ǀ CT: 10 (24%) ǀ TT: 2 (5%)	0.5
TF rs1130459	CC: 9 (17%) ǀ CT: 26 (48%) ǀ TT: 19 (35%)	CC: 4 (9%) ǀ CT: 21 (50%) ǀ TT: 17 (40%)	0.6
TF rs12769	CC: 20 (38%) ǀ CT: 28 (53%) ǀ TT: 5 (9%)	CC: 12 (29%) ǀ CT: 23 (55%) ǀ TT: 7 (17%)	0.5
